# Effect of fiber-reinforced direct restorative materials on the fracture resistance of endodontically treated mandibular molars restored with a conservative endodontic cavity design

**DOI:** 10.1007/s00784-024-05720-4

**Published:** 2024-05-16

**Authors:** Merve Nezir, Beyza Arslandaş Dinçtürk, Ceyda Sarı, Cemile Kedici Alp, Hanife Altınışık

**Affiliations:** 1 Department of Restorative Dentistry, Faculty of Dentistry, Gazi University, Emek, Ankara, 06510 Turkey; 2 Department of Restorative Dentistry, Faculty of Dentistry, İstanbul Medipol University, İstanbul, Turkey

**Keywords:** Bulk-fill, Fiber, Access cavity, Endodontic treatment, Resin composite

## Abstract

**Objective:**

This study aimed to evaluate the fracture strength of teeth restored using fiber-reinforced direct restorative materials after endodontic treatment with a conservative mesio-occlusal access cavity design.

**Materials and methods:**

A total of 100 extracted intact mandibular first molars were selected and distributed into a positive control group where teeth left intact and the following four test groups comprised of teeth with conservative mesio-occlusal access cavities that had undergone root canal treatment (*n* = 20/group): access cavity without restoration (negative control), bulk-fill resin composite with horizontal glass fiber post reinforcement, fiber-reinforced composite with bulk-fill resin and bulk-fill resin composite. Following thermocycling (10,000 cycles), fracture resistance was measured using a universal testing machine. Statistical analyses (one-way analysis of variance and the Tamhane test) were performed, and statistical significance was set at *p* < 0.05.

**Results:**

Groups with minimally invasive access cavities had lower fracture strength than intact teeth, regardless of the restoration material (*p* < 0.05). Fiber-reinforced composite groups demonstrated higher fracture strength than bulk-fill resin composite alone (*p* < 0.05). Fracture types varied among groups, with restorable fractures predominant in the fiber-reinforced composite groups.

**Conclusion:**

This study suggests that using fiber-reinforced composite materials, especially in combination with bulk-fill resin composites, can effectively enhance the fracture strength of endodontically treated teeth with conservative access cavities. However, using only bulk-fill resin composite is not recommended based on the fracture strength results.

**Clinical significance:**

When teeth that undergo endodontic treatment are restored using a conservative access cavity design and fiber-reinforced composite materials, especially in combination with bulk-fill resin composites, the fracture strength of the teeth can be effectively increased.

## Introduction

The long-term success of endodontic treatment is affected by an endodontic access cavity design that preserves the tooth structure as much as possible, the quality of root canal treatment procedures, the type of ideal restoration to compensate for the loss of coronal tooth structure and the application technique [1, 2]. For decades, traditional endodontic cavity designs, whose outlines are standardized for each tooth type, have remained virtually unchanged, completely removing the pulp chamber roof to detect root canals and creating straight-line pathways into the canals to increase the effectiveness of instrumentation [3–5]. However, such procedures remove most of the tooth structure, including peri-cervical dentin [3]. Positioned roughly 4 mm above and 6 mm below the alveolar bone crest, the peri-cervical dentin is recognized as a vital component for the overall longevity and proper functioning of the tooth [6], playing a critical role in distributing forces from the occlusal aspect to the root(s) [7]. Increasing cuspal flexure by removing peri-cervical dentin during traditional endodontic cavity preparation increases the stress on the crown and root surfaces of teeth, which may increase the possibility of fracture in endodontically treated teeth when subjected to functional loads [8–10]. Clark et al. [11] suggested that preserving a 0.5–3 mm pulp chamber roof is the most secure approach to avoid causing harm to this dentin.

Therefore, with advancements in magnification and imaging techniques as well as endodontic instruments, conservative endodontic cavity designs, which aim to protect the remaining tooth tissue during access cavity preparation, have been developed as an alternative to traditional endodontic cavity designs [12]. In conservative endodontic cavity designs, the removal of restorative materials instead of enamel or dentin and the removal of occlusal structures instead of peri-cervical dentin are preferred. This approach preserves parts of the pulp chamber’s roof while safeguarding the peri-cervical dentin, which helps maintain the mechanical stability of the tooth, ultimately prolonging its lifespan and enhancing its functionality [6, 7, 13]. However, conservative cavity designs may limit proper irrigation and instrumentation, obturate root canals, and lead to more errors during endodontic procedures [14].

Several studies have proposed new, more conservative and ultraconservative approaches for preparing endodontic access cavities, such as the conservative, Ninja and truss access designs [12, 15]. The Ninja endodontic access cavity design consists of opening a small hole from the central fossa or the deepest part of the occlusal surface, allowing the clinician to locate and access all canal orifices [15]. The truss endodontic access cavity design uses direct access from the occlusal surface to each canal orifice and maintains a dentin bridge between the canal orifices [16]. In addition, some new conservative approaches for access cavity preparations have also been introduced, as sometimes the tooth already has restorations or has some caries patterns in which traditional or conservative access cavity preparation cannot be achieved (For example, as in this study, the mesial canals are accessed from the mesio occlusal cavity, which is opened due to caries or old restoration in the mandibular first molar tooth. For the distal canal, a truss endodontic access cavity design is used to provide direct access from the occlusal surface) [17, 18]. A recent study showed that a truss endodontic access cavity could increase the fracture strength of endodontically treated teeth compared with other conservative endodontic cavity designs and traditional endodontic access cavity design [4].

In the literature, there are treatment options for the restoration of endodontically treated teeth, such as post-core and total or partial crowns, and advancements in adhesive technologies provide the opportunity to create conservative and aesthetically pleasing dental restorations entirely with composite resin materials [19, 20]. A retrospective study reported that the 5-year survival rate of severely damaged endodontically treated molars restored directly with composite resin was 18%, while the cumulative survival rate increased to 78% when the maximum amount of dental tissue was present [21]. For endodontically treated posterior teeth exhibiting conservative endodontic cavity designs, the most suitable access restoration consists of a direct restoration using a resin composite due to its high bond strength to the dental substrate [22]. The comparison of fracture resistance of endodontically treated upper premolar teeth after restoration of the two-walled access cavity preparation using direct composite, indirect composite and computer aided design/computer aided manufacturing CAD/CAM ceramic inlays showed that composite resins have the highest fracture resistance [23].

To strengthen direct composite resin restorations, glass fiber can be added horizontally to the coronal structure, or fiber-reinforced resin composites can be used to increase the fracture resistance of the core structure due to their suitable physical properties [24, 25]. Using glass fibers in the restoration of access cavity restoration enhances the flexural properties of composite resin, providing effective force transmission and high fracture resistance [26]. EverX Posterior has been introduced to the market as a dentine replacement material, featuring E-glass fibers in its composition [27]. The manufacturer claims that using EverX Posterior (GC, Tokyo, Japan) in stress-bearing areas enables the restoration to exhibit dentin-like stress management, preventing the progression of formed cracks [28]. High-tech production of this special component composite has allowed us to provide the high fracture resistance of composite restorations, even in large posterior cavities [29].

The introduction of bulk-fill resin composites has recently gained attention due to their translucency, allowing for an induced degree of conversion rates and the ability to polymerize effectively at a thickness of 4–5 mm [30]. An increased degree of conversion allows the material to have better mechanical properties and enables its use in stress-bearing areas, especially in post endodontic restorations [31].

Information on the effects of minimally invasive access cavity design, optimal restoration techniques and the use of optimal material is limited. Hence, the present study aimed to compare the fracture strength of teeth restored with a bulk-fill resin composite, EverX Posterior and a glass fiber post restorative system after endodontic treatment, utilizing a conservative endodontic cavity design.

The null hypothesis of this study was that the type of restorative material and technique used would not affect the fracture strength or fracture type of endodontically treated teeth with a conservative endodontic cavity design.

## Materials and methods

A total of 100 intact human mandibular first molars extracted for periodontal reasons were used for this study. All teeth were free from caries, defects, restoration and cracks and were used in compliance with ethical guidelines of Gazi University Faculty of Dentistry Clinical Research Ethics Committee (ethical protocol no. 2021.19/6). Plaque, attached periodontal tissues and calculus deposits were removed by hand-scaling and then stored in 0.1% thymol solution until use.

The bucco-lingual and mesio-distal dimensions of the teeth were measured using a digital caliper (Insize 1112 − 150, Insize, Jiangsu, China). The teeth were divided into five groups so that there was no difference in the one-way analysis of variance (ANOVA) test between bucco-lingual and mesio-distal widths (*p* > 0.05) [32, 33]. The materials used in the study and their contents are listed in Table 1.

**Table 1:** The composition of the materials used in the study

Material	Manufacturer	Composition
Scotchbond Universal Etchant (Orthophosphoric Acid Gel)	3 M ESPE, St. Paul, MN, USA	35% Orthophosphoric Acid
Fiber Post	Cytec Blanco, Hannerkratt, Germany	Glass Fiber
Clearfil S^3^ Bond Universal (Universal Adhesive Resin)	Kuraray, Osaka, Japan	10-MDP, Bis-GMA, HEMA, hydrophobic dimethacrylate, camphorquinone, ethanol, water, silanated colloidal silica
Filtek One Bulk Fill Restorative (FOB) (Bulk-Fill Resin Composite Restorative Material)	3 M ESPE, St. Paul, MN, USA	Monomers: AUDMA, DDMA, UDMA Fillers: Ytterbium trifluoride (YbF3), zirconia filler, silica filler (76% by weight, 58% by volume, 0.004–0.01 μm size)
EverX Posterior (EXP) (Fiber-Reinforced Resin Composite Restorative Material)	GC, Tokyo, Japan	Bis-GMA, PMMA, TEGDMA, short E-glass fiber filler, barium glass filler (74.2% by weight, 53.6% by volume)

Abbreviations: 10-MDP, 10-methacryloyloxydecyl dihy- drogen phosphate; Bis-GMA, Bisphenol A diglycidylmethacrylate; HEMA, 2-hydroxyethyl methacrylate; AUDMA, Aromatic dimethacrylate; DDMA, 1,12-dodecane dimethacrylate; UDMA, Urethane dimethacrylate; PMMA, polymethyl methacrylate; TEGDMA, triethyleneglycol dimethacrylate

Before preparing the cavities in the teeth, the distance between the cusps was measured using the digital caliper. To prepare a conservative access cavity, digital radiography was used to determine the borders of the access cavity according to the dimensions of the teeth. Then, the canal entrances were confirmed by considering the length of the periodontal probe according to the notches of the periodontal probe on the radiograph. The gingival step of the cavity was designed to be 1 mm upon the cemento-enamel junction (CEJ).

The conservative mesio-occlusal (MO) endodontic access cavity was applied to all teeth except the control group (intact teeth) using a straight fissure carbide bur (Hicare Medical Co. Ltd., Guangzhou, China) using a high-speed handpiece under water cooling and all Cavo surface margins prepared at 90° with internal line angles rounded. The bur was changed after every five cavity preparations, and cavity dimensions were measured during the preparation using a digital caliper to ensure standardization.

Following the endodontic access cavity, the working length was determined using #10 K-files (Shenzhen Perfect Medical Instruments Co. Ltd., Shenzhen, China), and the root canals were instrumented using ProTaper rotary files (Endoart, İstanbul, Turkey) up to sizes F3 with the crown-down method. During instrumentation, the root canals were irrigated with 1 mL of 2.5% NaOCl before each file was introduced into the canal and finally with distilled water. The canals were dried with paper points (Diadent Group International, Almere, the Netherlands) and obturated with gutta-percha cones and an AH Plus sealer (Dentsply De Trey, Konstanz, Germany) using the single cone technique. Excess gutta-percha cones were cut 1 mm apically from the canal orifices with a gutta cutter (C-Blade, Coxo, Guangdong Province, China) and covered with resin-modified glass ionomer cement (Ketac Cem Plus, 3 M, St. Paul, MN, USA). All teeth were examined after root canal treatment using periapical radiography to ensure accurate root canal treatment.

To simulate the clinical conditions, each specimen was embedded in a block of self-curing acrylic cylinders at a level of 2.0 mm below the CEJ. The periodontal ligaments were simulated using a base plate wax at a 0.3 mm (Efes Dental, Bursa, Turkey). The distal cavity was determined and standardized according to the distal marginal ridge thickness and periapical radiography. On the mesial side, the distance between the gingival margin and the CEJ was prepared at 1 mm. Care was taken to ensure a thickness of approximately 2 mm between the buccal and lingual walls and the interproximal cavity walls using the digital caliper. The buccolingual size of the cavity was 4 mm, and this width was measured above the cavities [34].

Clearfil S3 Bond Universal (Kuraray, Osaka, Japan) was applied using total etch mode with 35% orthophosphoric acid gel (Scotchbond Universal Etchant, 3 M ESPE, St. Paul, MN, USA). Acid gel was applied and left on enamel for 30 s and on dentin for 15 s, before being rinsed with water and gently dried with air to create a moist dentin surface. Clearfil S3 Bond Universal (Kuraray, Osaka, Japan) was applied using a micro-brush for 20 s and light-cured for 10 s with an LED light curing unit (D-Light Pro, GC, Leuven, Belgium).

**Fig. 1:**
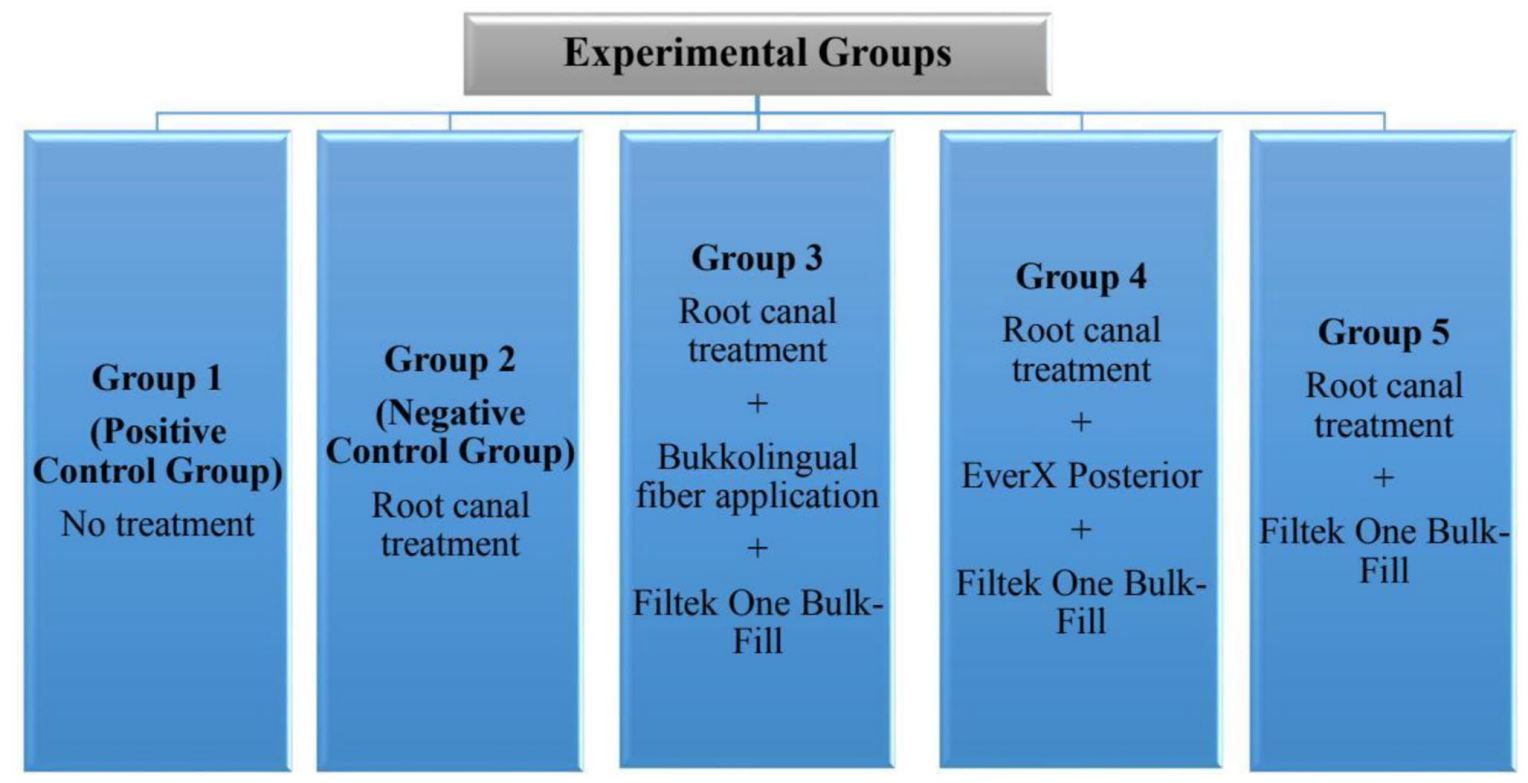
Study Design

**Fig. 2:**
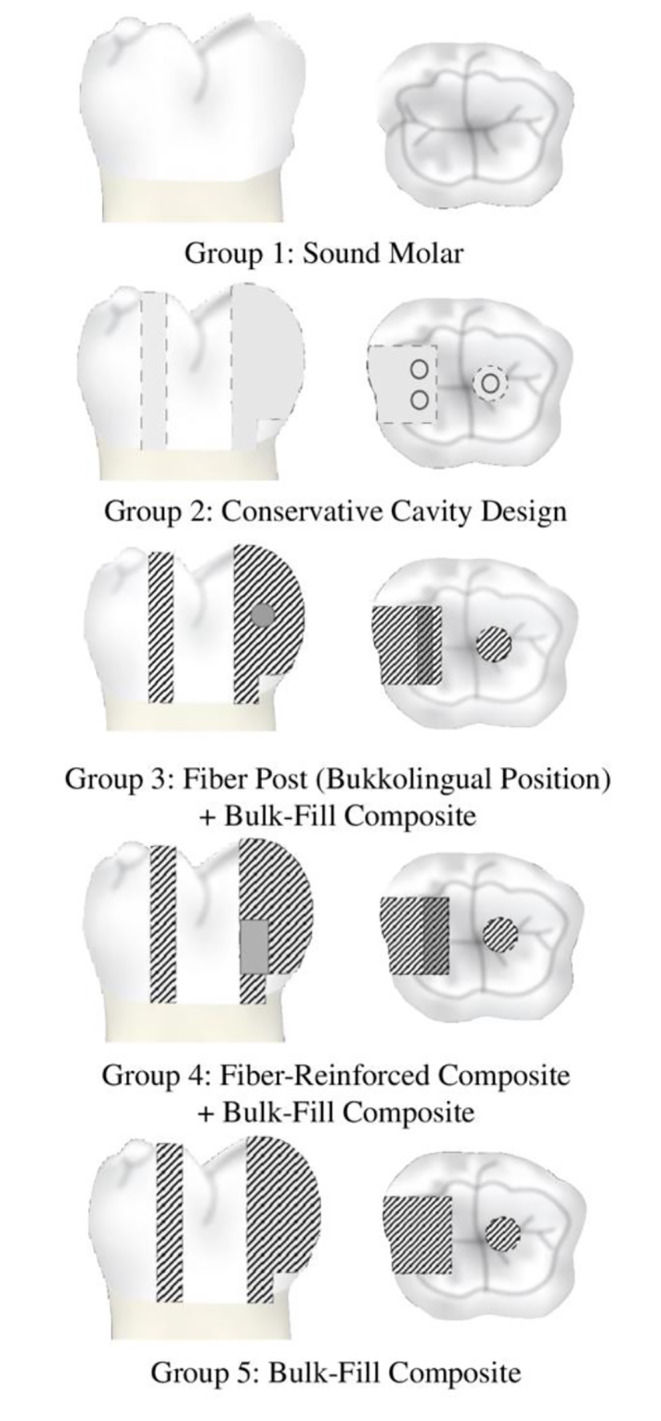
Study groups

Upon completion of root canal treatment, the restorative steps for each group were as follows (Figs. 1 and 2):

- Group 1 (positive control group): Intact teeth without any cavity preparation were used as the positive control.
- Group 2 (negative control group): The mesial endodontic access was prepared, and coronal restoration was not applied.
- Group 3: A 4 mm glass fiber post (Cytec Blanco, Hannerkratt, Germany) was cut with a diamond fissure bur. The post was then fixed to the buccal and lingual walls of the cavity with a flowable resin composite (Filtek Ultimate Flowable, 3 M Espe, St. Paul, MN, USA). For this purpose, a flowable resin composite was applied to both ends of the post and adapted to the buccal and lingual cavity walls in the middle one-third of the cavity. The overflowing flowable resin composite was removed using a sond. Then, the flowable resin composite was polymerized at the connection points of the post with the cavity using an LED light device for 20 s. Then, bulk-fill resin composite (Filtek One Bulk Fill Restorative, FOB; 3 M ESPE, St. Paul, MN, USA) was applied in 4-mm layers for the restoration of the entire cavity and polymerized on the occlusal, buccal and lingual sides for 10 s using an LED Light Device.
- Group 4: A 4-mm thick fiber-reinforced composite (EverX Posterior, EXP; GC, Tokyo, Japan) was applied to the first half of the cavity and polymerized with an LED light device for 20 s. Then, a 4-mm thick bulk-fill resin composite (Filtek One Bulk Fill Restorative, FOB; 3 M ESPE, St. Paul, MN, USA) was applied to the upper part of EverX Posterior, and the restoration was completed by polymerizing the occlusal, buccal and lingual sides for 10 s using an LED Light Device from.
- Group 5: Coronal restoration completed with FOB using the bulk technique.

The preparation and restoration of all specimens were performed by the same operator. Teeth were stored in distilled water for 24 h at 37 °C to prevent dehydration of the teeth and complete the post polymerization. They were then thermocycled at 5 °C and 55 °C for 10,000 cycles with a 30-s dwell time (MTE-101, MOD Dental, Ankara, Turkey).

To determine fracture resistance, a 5 mm stainless steel spherical tip mounted on a universal testing machine (Shimadzu IG-IS, Tokyo, Japan) and a 45° oblique compressive load were applied to the central fossa of the teeth at a crosshead speed of 1 mm/min until a fracture occurred. The maximum load before fracture was recorded in Newtons (N). The fractured specimens were examined by two different operators, indicating the fracture mode as restorable (fracture above the CEJ or within 1 mm apical to the CEJ) or unrestorable (fracture more than 1 mm apical to the CEJ) [35, 36].

### Statistical analysis

The sample size was calculated with G*Power software (version 3.1.9.6, Franz Faul, University of Düsseldorf, Germany). The effect size was 0.4, and the type 1 error (α) was 0.05. The analysis power was 0.80, which secured a minimum of 20 teeth per tested group.

The Shapiro-Wilk test showed that the fracture strength values were normally distributed (*p* > 0.05). The fracture strength of molars between the five groups was compared using one-way ANOVA, followed by the Tamhane post hoc test, at a significance level of 0.05. All statistical analyses were performed using SPSS 20.0 software (IBM Corporation Software Group, Armonk, NY, USA).

## Results

The mean and standard deviation of fracture resistance are displayed in Table 2.

**Table 2:** Mean fracture strength values and standard deviations by groups

	Minimum (*N*)	Maximum (*N*)	Mean (*N*)	Standard Deviation
**Group 1**	2172.115	2596.275	2384.195^a^	212.08
**Group 2**	1233.116	1617.724	1425.42^b^	192.304
**Group 3**	1758.918	2153.378	1956.148^c^	197.23
**Group 4**	1724.81	2289.49	2007.15^c^	282.34
**Group 5**	1276.67	1819.69	1548.18^b^	271.51

*Same uppercase letter indicates no significant difference (p > 0.05)

Group 1, the positive control group (2384.195 N), had a significantly higher fracture strength than the other experimental groups (*p* < 0.05).

Although there was no statistically significant difference between the fracture strengths of Group 5 restored with bulk-fill resin composite (1548.18 N) and Group 2 with only the access cavity prepared (1425.42 N) (*p* > 0.05), both groups exhibited statistically significantly lower fracture strength values than the other treatment groups (*p* < 0.05).

Groups 3 (1956.148 N) and 4 (2007.15 N) exhibited statistically similar fracture strengths (*p* > 0.05), and these groups demonstrated statistically significantly higher fracture strength values than all other groups except the positive control group (Group 1; *p* < 0.05).

**Graph 1:**
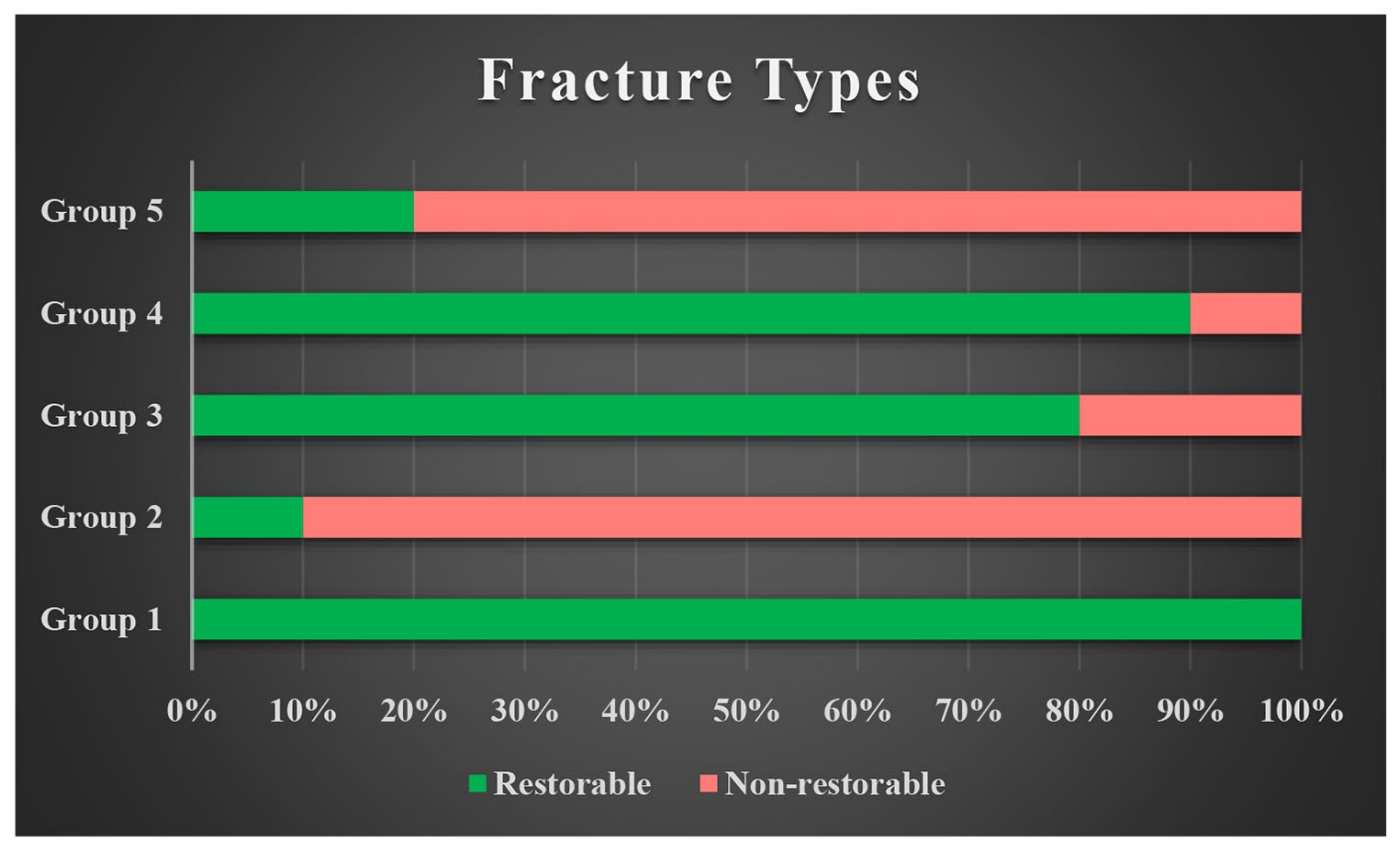
Distribution of fracture types in groups

The frequencies of fracture types classified as restorable or unrestorable are shown in Graph 1. Regarding the failure mode, the highest percentage of restorable fractures was observed in Groups 1 (positive control group: 100%), 3 (80%) and 4 (90%), while the lowest percentage was observed in Group 2 (negative control group: 10%).

## Discussion

With the rapid development of materials and techniques, determining the optimum technique is a complicated decision. According to current literature, direct resin composites are suggested for restoring teeth that have undergone root canal treatment due to their preservation of more hard tooth tissue, pleasing aesthetic qualities, sufficient mechanical strength, and intracoronal reinforcement [37, 38]. The concept behind minimally invasive direct restorations is to conserve as much tooth structure as possible, ensuring a durable bonded restoration that allows for future options in case of failure [39]. In addition, since the importance of coronal seal has been demonstrated in the literature, to prevent coronal microleakage, the final restoration should be started as soon as possible after root canal treatment, better still at the same visit as endodontic treatment [40]. Delaying permanent restoration and leaving the root canal treated tooth with temporary restorations increases the risk of periapical recontamination and future failure [41]. In their meta-analysis study, Kashi et al. examined the survival rate of direct and indirect restorations of root canal-treated teeth and did not detect a significant difference between the two restoration types. Hence, our samples were restored utilizing quick and cost-effective direct composite materials to investigate the impact of coronal restoration within conservative endodontic cavities [42].

Etching of dental hard tissue is performed to enhance surface roughness by dissolving hydroxyapatite minerals within the structure, eliminating smear plugs within dentinal tubules and aims to facilitate optimal adhesive bonding. Additionally, although phosphoric acid etching aims to aid adhesive diffusion by dissolving enamel rods, the resulting thick, HAp-free hybrid layer formed on dentin is highly susceptible to degradation over time [43]. Self-etch adhesives containing acidic monomers dissolve both the smear layer and the underlying dentin structure, thus obviating the requirement for a separate acid application. In addition to being user-friendly, they reduce post-operative sensitivity without decreasing bond strength, thanks to their tendency to cause superficial demineralization of dentin and partial occlusion of the dentinal tubules in the smear layer [44]. Pretreating the dentin surface with a deproteinizing agent, such as sodium hypochlorite (NaOCl), hypochlorous acid (HOCl) or papain solution, can be used with self-etch adhesives as a novel technique aimed at enhancing resin/dentin hybridization [45, 46]. Also bonding procedures for coronal and radicular dentin are similar, but variations exist in dentin structural components, mineral composition, and properties based on location and physiological changes due to aging and pathology [47]. Kusumasari et al. compared the push-out bonding strenght of self-etch and total-etch modes of universal adhesive systems on apical and coronal dentin surfaces and associated the low bond strength of etch and rinse systems with the exposure of a deeper collagen-fibril network after excessive acid exposure [48].

In contrast to conventional self-etch adhesives, Scotchbond Universal possesses a low pH level and incorporates 10-MDP and HEMA molecules in its composition, thereby enhancing adhesion capabilities [49]. Innovatively, 10-MDP monomers interact with calcium without inducing significant demineralization on dentin and enamel surfaces. This interaction is aimed at supporting bond strength by facilitating the formation of MDP-Ca salts [50]. Complementarily, the HEMA molecule within the adhesive causes adhesion to dental tissues by chemically engaging with hydroxyapatite. This process effectively enhances dentin wettability through the adhesive’s inherent hydrophilic nature [51]. Together, these mechanisms contribute to the adhesive’s efficacy in dental applications, offering improved bonding capabilities and stability.

Coronal restoration of endodontically treated teeth that are at greater risk of fracture is quite challenging. Endodontically treated maxillary second premolars and mandibular molars are the most fractured teeth during the natural masticatory process [52]. The present study included mandibular first molars due to their high extraction frequency and susceptibility to fractures [53]. It is crucial to simulate the physiological environment in clinical practice in in vitro studies of dental materials. The thermal cycle is a widely used procedure for simulating the aging of restorative materials [54]. The lack of standardization makes the decision between the thermal aging protocols challenging. In our study, we applied the 10,000-cycle protocol of Gale et al., which is equivalent to 1 year of clinical function, as it is a widely accepted method [55].

In the intraoral complex, chewing forces are transmitted to the root surface along the long axis of the tooth through the periodontal ligament [56]. Continuous forces can lead to PDL compression, which may result in minimal tooth movement. Soares et al. reported that the resin material in which the samples were embedded did not affect the fracture strength results, but imitating the periodontal ligament affected the results [57]. Therefore, we used modeling wax to imitate the periodontal ligament in our study. The occlusal load was directed at a 45-degree angle with a universal test machine following the approach of Plotino et al. to accurately replicate the eccentric forces in the chewing movements [58]. The choice of load direction is important for replicating the physiological chewing movement and causing fracture of the restoration-tooth complex at lower loads [18].

Previous studies have shown that the amount of structure left after cavity preparation is the main factor in the long-term survival rate of root canal-treated teeth [59, 60]. The traditional endodontic cavity preparation approach, characterized by excessive tissue removal from the structure, exposes crown and root surfaces to induced stress from functional loads, consequently elevating the risk of fracture susceptibility [61]. Minimally invasive endodontics emphasize the minimal removal of tissue and the preservation of intact hard dental tissue at every step, spanning from access cavity preparation to root canal instrumentation [62]. Minimally invasive cavity preparation aims to protect peri-cervical dentin and partial deroofing in root canal-treated teeth [63]. The amount of peri-cervical dentin functions as a stress distributor and is crucial for the distribution of oclusal load to the radicular portion of the tooth [64]. Using finite element analysis, Zelic et al. found that access cavity design preparation had the greatest influence on tooth strength in endodontically treated teeth [65]. Despite the advancement in material science and the with the concept of minimally invasive procedure, all groups with minimally invasive access cavities in our study had significantly lower fracture strengths than intact teeth, irrespective of the material used (*p* < 0.05). For the purpose of strengthening teeth, the use of reinforcement materials in the structure may be useful.

In the current literature, for endodontically treated teeth, fiber-reinforced resin composite materials are currently the recommended option [66]. In this study, the fracture strength of the fiber-reinforced groups was significantly higher than that of the bulk-fill resin composite groups (*p* < 0.05). Therefore, the null hypothesis of the study was rejected. Similar to our study, Mangoush et al. reported that fiber-reinforced resin composite restorative materials were more effective in strengthening structurally damaged teeth and increasing fracture strength than non-fiber-reinforced resin composite restorative materials [67]. EverX Posterior is a fiber-reinforced composite that is cured with light and is used as a dentine replacement material [68]. While the short fibers within the structure have a similar effect to the collagens in dentin, the combination of the dimethacrylate resin matrix and polymethacrylate chains known as semi-interpenetrating networks (semi-IPN) makes the structure fibrous [28]. The short E-glass fibers in the material’s structure are distributed multidirectionally, which enables the composite to exhibit anisotropic behavior under chewing forces [69]. Using the material under the conventional resin composite restoration in the pulp chamber increases the absorption of stress on the structure and the toughness of the restoration. Furthermore, EverX Posterior’s millimeter-scale short fibers stop cracks from spreading, minimizing the damaging impacts of internal cracks in the structure and promising fewer catastrophic fractures, as was found in our study [70].

No studies have detected an increase in the fracture strength of post endodontic restoration as a result of intraradicular post placement [71, 72]. Intraradicular post placement may weaken the root during post–space preparation and may also lead to procedural errors, such as strip perforation [73]. Another coronaradicular reinforcement method is to provide intracanal anchorage with composite resins without the use of fabricated fiber posts. Krastl et al. compared the fracture strength of intraradicular fiber, titanium post and composite anchorage and determined that composite anchorage had more repairable fractures and that fiber posts had a higher fracture load than titanium posts and composite anchorage [74]. Placing a fiber post in a horizontal position for reinforcement in teeth with remaining buccal and lingual walls is a fast, but not required, aesthetic restoration solution that requires a high skill set [75]. Broomberg et al. placed a single horizontal post in their study and found that the increase in fracture resistance was statistically significant by over 60% [76]. Bainy et al. compared the use of a horizontal fiber post and braided glass fiber in the structure to strengthen resin composites and found that both restoration types had a similar fracture strength [77]. In addition to the dentin-like elastic modulus of the fiber, the horizontal fiber post may have strengthened the coronal residual tooth structure by absorbing occlusal loads. Karzoun et al. compared the horizontal placement of fiber posts in endodontically treated teeth with conventional composites and found that the group of fiber posts with fracture strength doubled [78]. The groups in which the EverX Posterior was used in the pulp chamber and the horizontal fiber post placement groups had similar median values and statistically higher fracture strength. In their study, Naik et al. examined the fracture strength of endodontically treated teeth after restoration using horizontal fiber and fiber reinforced composites and found similar fracture strength in both groups, similar to our study [79]. The one possible explanation for the horizontal application of fiber post to achieve the fracture strength values as successful as fiber reinforced composites can be the reduction of the cusp deflection due to buccal and lingual anchoring [76]. Another explanation is that the fiber post, such as EverX posterior, has a similar elastic modulus to dentin. The strengthening effect of the fiber post is attributed to the plasticization of the polymer matrix by linear polymer chains of poly-methylmethacrylate within the cross-linked matrix. This process transfers stress from the polymer matrix to the fibers [80, 81].

Bulk-fill resin composites, introduced to the market in the early 2000s, have gained popularity with the promise of reducing chair time and allowing placement of up to 5 mm in large cavities with the bulk technique [82]. Although mechanical behavioral successes are still controversial in the literature, there are also many studies with successful results [38–40]. Optimally, direct composites should be applied with an incremental technique to reduce polymerization shrinkage and microleakage in restorations with large cavities, such as teeth with a root canal [83, 84]. Curently, bulk-fill composites exhibit higher translucency compared to conventional composites, incorporating alternative photo-initiator systems and modified monomers to enable enhanced polymerization. Restoring the entire cavity with the bulk technique has not been recommended in previous studies because the effect of the c-factor is greater [85, 86]. In studies conducted in cavities with a similar low c-factor, as that in our study, no statistical difference has been detected between the incremental and bulk techniques in bonding performance and polymerization shrinkage [82, 87].

Due to their material-specific characteristics, the application of some bulk-fill resin composites as full-body restorations is possible, while others require a capping layer with a conventional composite for optimal mechanical performance [88]. EverX Posterior is a resin used for reinforcement in the core structure and is recommended by the manufacturer for capping due to its surface roughness and insufficient optical properties [89]. For this reason, in our study, we capped the groups using EverX with Filtek bulk-fill, as per the manufacturer’s instructions.

As demonstrated in various studies, the amount of filler in resin composites significantly contributes to increased fracture strength, with the suggested critical filler amount being above 60%. The tensile fatigue strength of composite resins with extremely low or high filler content (< 60% or > 80% by weight) has been shown to be significantly reduced [43]. Accordingly, the fracture strength of composite resins is also negatively affected. Filtek Bulk Fill exhibited the highest filler loading, at 58.5% by volume; this material also demonstrated significantly lower fracture strength values compared to the other groups tested. This can be explained by the weak bonding strength between the filler particle and the organic matrix or the presence of linear polymer chains of PMMA in the cross-linked matrix of Bis-GMA/TEGDMA, which might contribute to the increased fracture toughness of the composite resin. This is because the PMMA chains can act as plasticizers, making the polymer matrix more flexible and resistant to fracture [90]. In addition, El-Damanhoury et al. suggested that the flexural modulus of Filtek Bulk Fill is lower than that of EverX Posterior. This may explain why the material exhibits a lower elasticity than dentin after occlusal forces, leading to significantly high catastrophic fracture values [91].

Based on our results, the use of fiber-reinforced composite restorative materials in the restoration of endodontically treated teeth using a conservative endodontic cavity can effectively increase the fracture strength of the teeth.

## Conclusion

Since the fracture strength values of endodontically treated teeth restored with the combined use of fiber-reinforced resin composite material and bulk-fill resin composite material showed the values nearest to the fracture strength values of intact teeth, these materials can be preferred in the restoration of endodontically treated teeth. Thus, the use of only bulk-fill resin composite in the restoration of endodontically treated teeth is not recommended since the fracture strength values of teeth restored with only bulk-fill resin composite showed the nearest values to the negative control group. More clinical and laboratory studies are needed to investigate the success of materials for the restoration of endodontically treated teeth. Future research could involve long-term comparisons among intracoronal reinforced restorations and indirect techniques in endodontically treated teeth. Moreover, it is imperative to assess the fracture strengths of conservative access cavities in comparison to cusp coverage or traditional access cavities.

In this study, intraoral loads were applied only vertically, which is a dynamic and static structure. As a result, lateral forces that may occur clinically during clenching were not simulated. Additionally, compressive load testing is a frequently used method when investigating the behavior of restorations under certain conditions, but other tests such as finite element analysis, tensile tests and long-term clinical studies are also required to evaluate the performance of restorations. Only mandibular first molars were used in this study; therefore, these results may only be applied to that group of teeth. These limitations should be addressed in future research.

## Data Availability

No datasets were generated or analysed during the current study.

## Abbreviations

10-MDP: 10-methacryloyloxydecyl dihy- drogen phosphate
Bis-GMA: Bisphenol A diglycidylmethacrylate
HEMA: 2-hydroxyethyl methacrylate
AUDMA: Aromatic dimethacrylate
DDMA: 1,12-dodecane dimethacrylate
UDMA: Urethane dimethacrylate
PMMA: polymethyl methacrylate
TEGDMA: triethyleneglycol dimethacrylate
